# Obituary: Bruce McEwen

**DOI:** 10.1093/ijnp/pyaa012

**Published:** 2020-03-25

**Authors:** Michael J Meaney

**Affiliations:** 1 James McGill Professor, Faculty of Medicine, McGill University, Montreal Canada; 2 Director, Translational Neuroscience Programme, Singapore Institute of Clinical Sciences, Agency for Science, Technology and Research, Singapore

Bruce McEwen was born in Fort Collins, Colorado January 17^th^ 1938. He died suddenly following a brief illness on January 2^nd^ of 2020. Between those dates he forever changed way we think of the relation between our brain and our circumstance. He will be forever remembered for an unprecedented ability for integrative science that bridged the biological and social sciences, and rendered neuroscience relevant to our discussions of health policy and social justice. Bruce had an astoundingly productive career of near 1400 publications, countless awards, and prominence (he was a past President of the Society for Neuroscience). But for those of us fortunate to know him well, the dominant memory is of Bruce’s good humor, generosity, modesty and humanity. To know him, even casually, was to understand how we feel that Bruce McEwen the person transcended Bruce McEwen scientist - hard as that is to imagine.

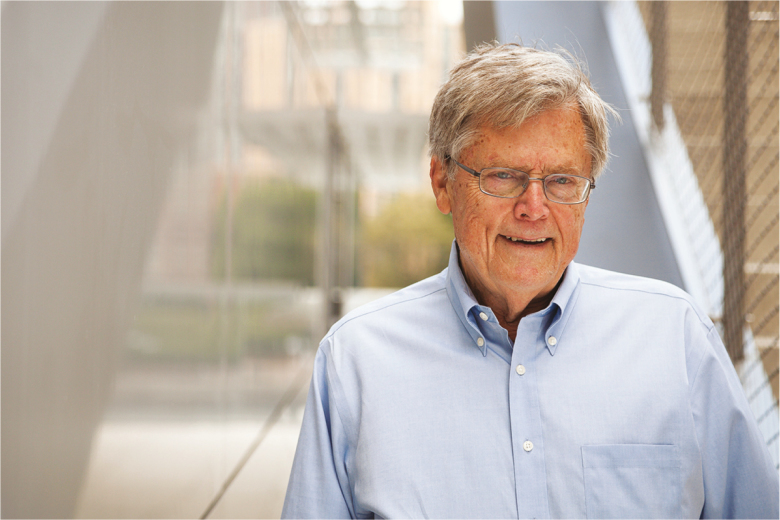



Bruce was raised in Ann Arbor, Michigan and pursued a bachelor’s degree in chemistry from Oberlin College and a Ph.D. in cell biology from Rockefeller University studying energy metabolism in the cell nuclei in relation to RNA synthesis. Bruce’s early career path was influenced by the fascination of his mentor, Alfred Mirsky, with the potential for environmental regulation of gene expression, a topic that would remain at the core of McEwen lab science. Following post-doctoral studies at the University of Gothenburg, Sweden, he became an Assistant Professor at Rockefeller in the team of Neal Miller, one of the leading figures of the day in psychology, who was captivated by the potential for research in “behavioral medicine.” Bruce would often recount how these and other experiences had forged the path by which he became a “molecular sociologist.” Bruce’s training provided a vision for the integration of cell and molecular biology with the social sciences and, ultimately, the study of health across the lifespan. It was Bruce’s science that brought that vision scientifically to life. Indeed, even more impressive than the volume of Bruce’s outstanding science was its breadth and impact.

The legacy of the McEwen lab at The Rockefeller University- the emergence of the neuroscience of stress- derives from Bruce’s discovery of corticosteroid receptors in the brain. This finding provided a mechanism whereby the classic stress-related endocrine signal could act in the brain. And not just the hypothalamus – the primary region identified in Bruce’s analyses was the hippocampus. This finding would ultimately provide the basis for a biological unification of the “body” and “brain” in the organism’s response to environmental conditions.

But not without some dissention. Ron de Kloet, an emerging star in steroid biochemistry, wrote to Bruce that he had been unable to replicate the corticosteroid retention in the brain using dexamethasone, a potent synthetic ligand for the glucocorticoid receptor. Many would have dismissed this as an anomaly, technical artifact, etc. Not Bruce. His response was to invite Ron to spend time at Rockefeller. The result was a series of landmark papers on the biochemistry of corticosteroid receptors. As an aside, Ron and his lab latter showed that dexamethasone does not readily gain access to the brain, thus explaining the initial discrepancy. The story reveals much about Bruce’s character.

Despite the ultimate impact of the neural glucocorticoid receptor system, much of the research in Bruce’s lab in the 1970’s focused on estrogen and progesterone receptors, and the topic of sexual differentiation. Bruce’s lab was *the* site for the development of assays that successfully extracted steroid receptors from chromatin in isolated brain regions, thus allowing for an identification of region-specific, nuclear steroid signaling. This period featured the identification of the mechanisms for the sexual differentiation of the neural control of gonadal function and behavior revealed by a stream of remarkable research fellows under Bruce’s tutelage. The subsequent research on the lifelong effects of gonadal hormones on synaptic plasticity through both genomic and non-genomic mechanisms was yet another fascinating dimension to the orchestration of synaptic remodeling by steroid hormones.

The emphasis on stress and corticosteroid receptor signaling in the brain re-emerged brilliantly in the early 1980’s as Robert Sapolsky launched his remarkable career under Bruce’s supervision. The resulting research revealed a cascade whereby chronic stress produced a loss of hippocampal corticosteroid receptors, a resulting failure of HPA regulation, and the risk for glucocorticoid-induced neurotoxicity in the vulnerable, aged brain (“the glucocorticoid cascade hypothesis”). The science of glucocorticoid cascades had begun with identification of hippocampal corticosteroid receptors as a fundamental signal regulating hypothalamic-pituitary-adrenal (HPA) responses to stress. This stream of research reflected a major commitment in the McEwen lab to move beyond molecular biology to understand the broad physiological and behavioral significance of neuroendocrine signals. This line of research ultimately revolutionized our understanding of the adult brain. Through to the 1990’s we imagined a brain as plastic only during early periods in development and thereafter resolutely fixed in connectivity, save for the ravages of later life. The McEwen lab, instead, defined an adaptive brain in which dynamic variation in synaptic plasticity of the adult brain anchored the process of adaption to a range of environmental challenges as well as the potential for pathological conditions associated with unremitting and severe chronic stress. Bruce’s commitment to an integrative body – brain physiology resulted in research that identified the importance of a wide range of stress-mediators including metabolic (e.g., IGF-1, leptin, ghrelin, insulin, etc) and immunologic as well as steroid signals in mediating the effect of stress on the plastic adult brain.

I was accepted as a Post-Doctoral Fellow into Bruce’s lab in the early 1980’s. My research plan emphasized the androgen receptor action and social behavior, a topic not previously (even remotely) included within the diverse range of topics under study in the McEwen lab. My rather orthogonal pursuits were never an issue; I suffered no lack of personal (and financial) support from Bruce. In Bruce’s view, you went to his lab to develop your career, not his.

While at Rockefeller Sapolsky and I independently contrived research examining the developmental regulation of glucocorticoid receptor expression in relation to HPA function. Robert would complete his own experiments and then begin ours. I, the more nocturnal, would complete my own experiments, and bring ours to closure much later. We were never quite sure when Bruce became aware of this activity – I suspect he was so all along. We finally met with him to lay out early findings and the rationale and greeted this venture with an obvious joy at seeing two fellows establish what became a flourishing collaboration within his dominion. These studies laid the groundwork for the research in my lab on the environmental regulation of epigenetic mechanism controlling gene expression, which featured the glucocorticoid receptor. Ironically (or not) the topic of environmental regulation of gene expression was the passion of Mirsky and of his inspiration of Bruce towards neuroscience. But the initial science derived from Bruce’s laboratory as a context for innovation and initiative; his passion was in providing opportunity – his joy was in seeing it taken.

With time Bruce McEwen became the most important voice in communicating the biological basis for the link between stress and health. A series of high-profile papers (e.g., *JAMA*, *The New England Journal of Medicine*, etc) alerted the medical community to the evidence for the pathways linking stress and multiple non-communicable diseases. It is impossible to overstate the importance of this contribution for clinical medicine, where the concept of stress had until that time been largely unable to find a foothold in medical training or practice for what was thought to be a lack of biological plausibility.

Bruce then went beyond the documentation of existing evidence. Bruce and Eliot Stelllar established the concept of allostasi*s,* which describe both the importance of stress-mediators in adaptation to chronic stress as well as the ultimate cost to health. Bruce and his collaborators then gave empirical life to allostasis by creating a cumulative index comprised of these stress mediators that that reflected chronic activation of immunologic (CRP), metabolic (hip:waist ratio, insulin resistance), cardiovascular (blood pressure) and endocrine (glucocorticoid) signals. The resulting metric provided a novel, integrative measure of chronic stress for epidemiologic analyses, including analyses of how conditions such as socio-economic status “get under the skin” to influence health. “Allostatic load” bridged physiology and the biomedical sciences with health psychology and sociology. Importantly the resulting studies were essential for economic analyses that informed at the level of policy and movements for social justice. Intervention and prevention programs targeting the disadvantaged in our societies have drawn their rationale (and often their funding) from the evidence-based studies of “toxic stress” inspired by science of the McEwen lab. These very tangible contributions were of enormous significance for Bruce with his deep sense of humanity and passion for social issues.

The discoveries of the McEwen lab and those that followed in their path have penetrated all disciplines of the social and biological sciences with profound impact on clinical medicine and population health. Our understanding of the biology of social economic status, racism, trauma, and social disintegration have extended the reach of neuroscience into the discourse on social justice. Intervention and prevention programs targeting the disadvantaged in our societies have drawn their rationale (and often their funding) from the evidence-based studies of “toxic stress” inspired by science that grew from the McEwen lab. The remarkable relevance of Bruce’s science for the issues our times reflect both his genius for transformative science as well as his passion for social justice.

I have dwelt on Bruce’s science, because to know his science is, to some extent, to know him. But Bruce is best revealed in his interactions and for the deep meaning he had for so many people – not the least of which were his trainees. One did not so much train in Bruce’s lab, as to grow and evolve. Bruce stated that the success of his offspring was of far greater satisfaction than any scientific accolade. It is a sentiment shared by, perhaps, a few scientists in the later stages of a successful career. For Bruce this was always the case. Bruce constantly encouraged independent thought and experimentation. This independence and the accompanying sense of “ownership” was the wave that carried us into our own careers. It is simply remarkable to realize how many eminently successful research programs were a continuation of the science begun while a McEwen Fellow. Bruce was able to inspire so many research ideas, without in the least depriving a fellow of the joy and satisfaction of their pursuit. Simply put, Bruce’s career did not advance from the contributions of his research fellows – it was very much the other way around.

Bruce’s modesty and generosity were legendary and extended well beyond the grounds of his beloved Rockefeller. If there is a common experience amongst Bruce’s progeny it is to visit an academic site and be regaled by accounts from students of the time Bruce dedicated to interacting with them so personally, to his attentiveness and appreciation of their research, and to simply marvel at the glow that each would exude upon the recounting. “Bruce McEwen spent 30 minutes going over my slides with me and asking questions and commenting so positively!” Imagine, Bruce McEwen! We can. He couldn’t. That was just Bruce. His passion for science, his humanity, his modesty. I have met so many of those graduate and undergraduate students who trace their direction and continued passion inspired by those encounters. The influence extended to young PI’s struggling with the daunting obstacles to launching a research career. Those moments that bring the torment of self-doubt coupled with the practical issues of gaps in technology and protocols. Personal approaches to Bruce were invariably met with encouragement and an invitation to visit the lab for a day, a week, a month, more. The McEwen lab became an exemplar of what we now refer to as “knowledge transfer.” Such McEwen “adoptees” came to populate all corners of neuroscience. Bruce pitched the most inclusive scientific tent. All were welcome. Bruce did not simply mentor people – he mentored an entire field of science. He nurtured a field of science because he nurtured people.

More impressive than even his remarkable scientific accomplishments is the manner in which Bruce achieved this success. Scientists are, in part, entrepreneurs by circumstance. We earn our livelihood through appointments linked to the success of our research programs measured by publications and research grants. And, as in any field, success associates with some degree of self-promotion and ego. How then to understand the incredible prominence of a scientist who was without ego and had not the slightest inclination for self-promotion? And Bruce did so through no calculated sense of ultimate self-gain – he simply loved science and its potential for common good. On January 2, 2020 the world of science lost a pioneering giant – more painfully, we lost a person who epitomized the best in our profession and in human character.

